# Trends in health-related economic inactivity by smoking status in England, 2013–2025: a population-based analysis

**DOI:** 10.1016/j.lanepe.2025.101419

**Published:** 2025-09-07

**Authors:** Sarah Jackson, Sharon Cox, Jamie Brown

**Affiliations:** Department of Behavioural Science and Health, University College London, London, UK

**Keywords:** Smoking, Economic inactivity, Labour force

## Abstract

**Background:**

Rising levels of economic inactivity due to long-term illness in the UK have important public health and socioeconomic implications. Smoking is a leading risk factor for many chronic conditions that impair work capacity and is strongly associated with socioeconomic disadvantage. We examined trends in health-related economic inactivity among working-age adults in England by smoking status from 2013 to 2025.

**Methods:**

We analysed data from 173,248 adults aged 18-64 y in the Smoking Toolkit Study, a nationally representative monthly survey. Health-related economic inactivity was defined as not being in paid work due to long-term illness or disability. Logistic regression modelled time trends adjusted for age and gender, overall, by smoking status (current, former, never), and by duration of abstinence among former smokers.

**Findings:**

Health-related economic inactivity more than doubled between 2013 and 2025 (2.5% [2.3–2.7%] to 5.5% [5.1–5.9%]; prevalence ratio = 2.21 [1.96–2.49]). Prevalence was consistently highest—and absolute increases over time were largest—among current smokers; reaching 11.3% [9.9–12.7%] in 2025, compared with 5.8% [5.0–6.6%] in former smokers and 3.3% [2.9–3.7%] in never smokers. This equates to ∼700,000 current smokers not in work due to ill health or disability in 2025, up from ∼390,000 in 2013. Among former smokers, inactivity declined with longer abstinence.

**Interpretation:**

Smoking is strongly associated with health-related economic inactivity, with absolute disparities widening over time. In early 2025, one in nine working-age adults in England who smoked was not in work due to long-term illness or disability. Reducing smoking prevalence and addressing its social determinants may contribute to tackling rising inactivity and improving labour market participation.

**Funding:**

10.13039/501100000289Cancer Research UK.

**Research in context**

**Evidence before this study**

We searched PubMed and Google Scholar for English-language studies published up to April 2025 using combinations of terms including “smoking”, “economic inactivity,” “long-term sickness,” “labour force participation,” and “disability-related unemployment.” Data from the UK’s Office for National Statistics (ONS) suggest that health-related economic inactivity among working-age adults has risen substantially in recent years, particularly since the COVID-19 pandemic. However, there is ongoing debate over the magnitude of this increase, with some independent analyses suggesting that response bias may have led to inflated estimates. Previous research has established that smoking contributes to numerous physical and mental health conditions that increase the risk of long-term sickness absence and unemployment. Smoking is also disproportionately concentrated in socioeconomically disadvantaged groups, among whom the burden of poor health and labour market exclusion is greatest. However, it is not clear whether recent trends in health-related economic inactivity have differed by smoking status, or how many working-age smokers in England are currently not in work due to ill health or disability.

**Added value of this study**

This study uses 12 years of nationally representative survey data from over 170,000 adults in England to examine changes in health-related economic inactivity among working-age adults, both overall and by smoking status. It confirms a substantial rise in inactivity due to long-term illness or disability between 2013 and 2025—particularly since the onset of the COVID-19 pandemic—and offers a lower, possibly more conservative, estimate than that reported by the ONS. It also provides up-to-date estimates of how this burden is distributed by smoking status. It shows that the prevalence of health-related economic inactivity is more than three times higher among current smokers than never smokers in England, with the absolute gap widening in recent years. As of early 2025, one in nine working-age smokers—approximately 700,000 people—were not in work due to long-term illness or disability. Among former smokers, the risk of inactivity decreases with time since quitting.

**Implications of all the available evidence**

Rising health-related economic inactivity poses a major challenge for public health systems and national economies. This study highlights smoking as an important and potentially modifiable contributor to this trend. These associations are embedded in broader patterns of structural inequality that shape both health behaviours and labour market outcomes. Policies aimed at reducing smoking uptake and increasing smoking cessation could support reductions in economic inactivity and improve labour market participation, but addressing this issue at scale will also require systemic action to improve the social, economic, and lived environmental conditions that maintain both smoking and work participation.

## Introduction

The UK is currently facing an employment crisis, with a significant number of working-age individuals out of the labour market due to ill health or disability.^1^ This has implications not only for the economy but also for public health, as being out of work is an important driver of health inequalities.^2^ According to the Office for National Statistics (ONS), a quarter of the working-age population (16-64 y) do not currently have a job,^3^ of whom approximately 2.7 million are not in work due to ill health.^1^ Around half of people with disabilities are not in paid work—a rate more than double that of the rest of the working-age population.^1^ Economic inactivity (defined as not actively looking for work or not available to start a job) appears to have increased since the COVID-19 pandemic^3^^,^^4^—driven largely by long-term physical and mental ill health and disability^5^—placing a strain on public services, reducing productivity, and contributing to stalls in economic growth. However, there has been some debate about the extent of the rise,^6^^,^^7^ with an independent report suggesting it may be partly due to bias in who has responded to the ONS’ Labour Force Survey (LFS; the main source of data on unemployment).^6^ Since the pandemic, the LFS has experienced a significant drop in response rates, raising concerns that it may no longer accurately represent the working-age population.^6^ Addressing the causes of work incapacity is a key government priority, as reflected in policies aimed at reducing long-term sickness-related unemployment and boosting workforce participation.^8^

Economic inactivity is a complex and multifactorial issue, shaped by the cumulative effects of social determinants such as poverty, education, geography, and access to healthcare and employment.^2^^,^^9^ Multiple factors likely contribute to increasing rates of health-related economic inactivity in recent years. The pandemic introduced new health burdens (such as long COVID), disrupted access to routine healthcare, and led to marked increases in psychological distress, particularly among younger adults.^10^, ^11^, ^12^, ^13^ While these are likely central to recent trends, there is also value in examining how longer-standing health behaviours—such as smoking—may intersect with these broader dynamics.

It is well established that smoking-related ill health contributes to economic inactivity.^14^, ^15^, ^16^ Smoking is a modifiable behaviour with severe disease outcomes (e.g., chronic obstructive pulmonary disease, cancers, and cardiovascular disease) that lead to and exacerbate disabilities, all of which can result in long-term work absence and economic disengagement.^17^, ^18^, ^19^ It is also associated with poor mental health,^20^, ^21^, ^22^ which is thought to have been an important driver of recent increases in long-term sickness absence.^4^^,^^23^ Smoking may also increase susceptibility to COVID-19 complications,^24^ raising questions about its role in shaping post-pandemic patterns of work incapacity. While smoking prevalence has declined in recent decades, it remains disproportionately concentrated in socioeconomically disadvantaged groups,^25^^,^^26^ among whom the burden of poor health and labour market exclusion is greatest.^27^ Structural inequalities may compound the effects of health behaviours like smoking, together contributing to poor health and exclusion from the labour market.

Estimates suggest smoking costs the economy in England in the region of £27.6 billion each year in lost productivity due to smoking-related unemployment, lost earnings, and premature mortality.^16^^,^^28^ Understanding the current importance of smoking in health-related economic inactivity can provide insights into how reducing smoking rates might support not only improved health outcomes, but also economic recovery and employment rates. It may also help to identify intersecting pathways through which health behaviours, social conditions, and structural disadvantage jointly shape labour market participation.

In this study, we examined national trends in health-related economic inactivity by smoking status between 2013 and 2025, using data from a large, representative household survey in England. Specifically, we asked: (i) how the age- and gender-adjusted proportion of adults economically inactive due to long-term illness or disability varied over time by smoking status (current, former, or never smoker), and (ii) among former smokers, how inactivity rates differed by duration of abstinence. These questions are important because they can help assess the extent to which smoking continues to be associated population-level risks of workforce exclusion—even as its prevalence declines—and whether sustained cessation is associated with improved employment outcomes. By characterising recent trends, we also aimed to estimate the number of current smokers in England who are economically inactive due to ill health, and describe the sociodemographic profile of this group to inform future interventions.

## Methods

### Pre-registration

The study protocol and analysis plan were pre-registered on Open Science Framework (https://osf.io/nygzr/). In addition to our pre-registered analyses, we explored differences in time trends by age group and provided descriptive data on psychological distress among those not in work due to long-term illness or disability.

### Design

Data were drawn from the Smoking Toolkit Study (STS), an ongoing monthly cross-sectional survey of a representative sample of adults (≥16 years) in England.^29^^,^^30^ The STS uses a hybrid of random probability and simple quota sampling to select a new sample of approximately 1700 adults each month. Interviews are held with one household member in selected geographic output areas until quotas are fulfilled. The quotas are based on factors influencing the probability of being at home (i.e., working status, age and gender). This hybrid form of random probability and quota sampling offers advantages over conventional quota sampling. Here, the choice of households to approach is limited by the random allocation of small output areas and rather than being sent to specific households in advance, interviewers can choose which households within these small geographic areas are most likely to fulfil their quotas. Therefore, unlike random probability sampling, it is not appropriate to record the response rate.

Data were collected face-to-face up to the start of the Covid-19 pandemic and via telephone from April 2020 onwards; the two modes show good comparability on key sociodemographic and smoking indices.^31^ Measures of sociodemographic characteristics and smoking status used in the survey have demonstrated good validity compared with other national surveys and sales data.^29^^,^^32^

### Participants

The present analyses used data from working-age respondents between March 2013 (the first wave to assess working status) and February 2025 (the most recent data at the time of analysis). Data were not collected from 16- and 17-year-olds between April 2020 and December 2021, so we restricted the sample to those aged ≥18 y for consistency across the time series. A total of 174,188 adults aged 18–64 years were surveyed in England between March 2013 and February 2025. We excluded 940 (0.5%) with missing data on working or smoking status, leaving a final sample of 173,248 participants.

### Measures

Health-related economic inactivity was operationalised as participants reporting that they are ‘not in paid work because of long-term illness or disability’, in response to the following question assessing their current working status: ‘Which of these applies to you?’ Participants were asked to select the option that best described them: Have paid job—full time (30+ hours per week); Have paid job—part time (8–29 h per week); Have paid job—part time (under 8 h per week); Not working—housewife; Self-employed; Full time student; Still at school; Unemployed and seeking work; Retired; Not in paid work because of long term illness or disability; Not in paid work for other reason; Refused.

Smoking status was assessed by asking participants which of the following best applied to them: (a) I smoke cigarettes (including hand-rolled) every day; (b) I smoke cigarettes (including hand-rolled), but not every day; (c) I do not smoke cigarettes at all, but I do smoke tobacco of some kind (e.g., pipe, cigar or shisha); (d) I have stopped smoking completely in the last year; (e) I stopped smoking completely more than a year ago; (f) I have never been a smoker (i.e., smoked for a year or more). Those who responded a-c were considered current smokers. Those who responded d-e were considered former smokers. Those who responded f were considered never-smokers.

For former smokers, we calculated the duration of abstinence in years (i.e., how many years ago a participant quit smoking). For those who quit in the past year (response d), this was assessed with the question: ‘How long ago did your most recent serious quit attempt start? By most recent, we mean the last time you tried to quit’ with response options coded as follows: In the last week [0.02 years]; More than a week and up to a month [0.05 years]; More than 1 month and up to 2 months [0.125 years]; More than 2 months and up to 3 months [0.21 years]; More than 3 months and up to 6 months [0.375 years]; More than 6 months and up to a year [0.75 years]. For those who quit more than a year ago (response e), it was calculated as the participant’s actual age minus the age when they stopped smoking. Duration of abstinence was analysed as a continuous variable (see Statistical analysis section).

Age was analysed as a continuous covariate for trend analyses and categorised as 18–24, 25–34, 35–44, 45–54, and 55–64 years for descriptive and age-stratified analyses. Gender was assessed with the question ‘Which of the following best describes how you think of yourself?’ with response options ‘male’, ‘female’, or ‘in another way’; we excluded the latter category for trend analyses due to the small number of participants (*n* = 657 across the entire period; mean [SD] = 8 [6] per month) but retained it for descriptive analyses. Region in England was categorised as North West, North East, Yorkshire and the Humber, West Midlands, East Midlands, East of England, South West, South East, and London.

Psychological distress was assessed using the Kessler Psychological Distress Scale (K6), which measures non-specific psychological distress in the past month (possible range 0–24)^33^^,^^34^; we coded scores ≤4 as no or low distress vs. 5–12 as moderate and ≥13 as severe distress.^33^^,^^35^ Between 2022 and 2025, this variable was only included in selected waves (January 2022–June 2023, January–March 2024, and January–February 2025), so analyses of distress are restricted to participants surveyed in these months.

### Statistical analysis

Data were analysed using R v.4.4.2. The STS uses raking (iterative proportional fitting)^36^ to weight the sample to match the population in England. The sociodemographic profile of the total population is determined each month by combining data from the UK Census, the Office for National Statistics mid-year estimates, and the annual National Readership Survey.^29^ The following analyses used weighted data. We excluded participants who did not report their smoking or working status (this was done after weights were calculated; no adjustments were made to account for these exclusions because they represented only a very small proportion of eligible participants [0.5%]).

#### Time trends

We used logistic regression to model time trends in the proportion of adults not in work due to long-term illness or disability between March 2013 and February 2025. To estimate changes over time among all working-age adults, we ran a model with time as the predictor. To examine differences by smoking status, we modelled the interaction between time and smoking status—thus allowing for time trends to differ between never, former, and current smokers. To examine differences among former smokers by duration of abstinence, we modelled the interaction between time and duration of abstinence. In an unplanned analysis conducted before peer review, we repeated these models stratified by age to explore differences in time trends by age group.

For all models, time (survey wave) was modelled using restricted cubic splines, to allow for flexible and non-linear changes over time while avoiding categorisation. We compared models with three, four, and five knots using the Akaike Information Criterion (AIC) and reported the best-fitting model for each outcome (selected as the model with the lowest AIC value or the simplest model within 2 AIC units; Supplementary Table S1). Duration of abstinence was modelled using restricted cubic splines, with three knots (placed at the 5th, 50th, and 95th percentiles; sufficient to model non-linear associations while avoiding overfitting). All models were adjusted for age and gender. Age was modelled as a continuous covariate; we considered using splines but the relationship between age and health-related economic inactivity was linear (Supplementary Fig. S1) so this was not deemed necessary.

To illustrate the extent of changes from the start to the end of the period within each subgroup, we reported the absolute percentage point change and the relative change (prevalence ratio [PR]) between March 2013 and February 2025, alongside 95% confidence intervals (CIs) calculated using bootstrapping (1000 replications). We used predicted modelled estimates to plot the age- and gender-adjusted proportion of never, former, and current smokers not in work due to long-term illness or disability over the study period.

Following peer review, we added an unplanned segmented regression analysis that tested the association of the onset of the COVID-19 pandemic with changes in the trend in health-related economic inactivity. We used log-binomial generalised additive models (GAMs, using the *mgcv* package in R) to model trends before the pandemic (underlying secular trend; coded March 2013 = 1 through February 2025 = 144) and the change in the trend (slope) after the pandemic onset (coded 0 up to March 2020 and 1 … *n* from April 2020 onwards, where *n* was the number of waves after the pandemic onset), overall and stratified by smoking status. A step-level change was not included as this was deemed implausible. To adjust for seasonality (month-of-year effects), we also included a variable reflecting the calendar month coded from January = 1 to December = 12; this variable was modelled using a smoothing term with cyclic cubic splines specified. We assumed a linear trend in log-prevalence before the announcement (i.e., the proportional change in prevalence month-on-month would be stable from March 2013 to March 2020). For ease of interpretation, we multiplied model coefficients by 12 to convert results from monthly to annual trends. The post-pandemic onset trend was derived by summing the pre-pandemic trend and the slope (change in trend after the pandemic onset) on the log scale and exponentiating the result to convert it to a risk ratio (RR). The 95% confidence interval (CI) for the post-pandemic onset trend was derived by combining the uncertainties of both components, calculating the standard error, and applying the usual CI formula before exponentiating.

#### Population size estimates

Using the Office for National Statistics’ (ONS) mid-year population estimates for England,^37^ we estimated the number of working-age current smokers not in work due to long-term illness or disability as of February 2025 (the final month of the study period). The calculation was as follows:

Estimatedsmokersnotinwork=totalpopulation×smokingprevalence×proportionofsmokersnotinworkduetoillness/disability

Where ‘total population’ is the number of adults aged 18-64 y in England (ONS 2023 mid-year estimates); ‘smoking prevalence’ is the proportion of working-age adults who currently smoke (STS; December 2024–February 2025 [we used data aggregated across the most recent three waves rather than just February 2025 to increase the sample size for this estimate]); and ‘proportion of smokers not in work due to illness/disability’ is the modelled estimate for February 2025 (STS).

For comparison, we also estimated the corresponding number of current smokers not in work due to long-term illness or disability in the first month of the study period (March 2013), using ONS 2013 mid-year estimates of population size, smoking prevalence in March–May 2013, and the modelled estimate for March 2013.

#### Sociodemographic profile

Finally, we used descriptive statistics to compare the profiles (i.e., sociodemographic characteristics) of current, former, and never smokers not in work due to long-term illness or disability. For this analysis, we restricted the sample to those surveyed between 2022 and 2025 (*n* = 2254), to provide up-to-date information while ensuring sufficient sample sizes. Within each smoking status, we calculated the proportions (with 95% CIs) of those not in work due to long-term illness or disability belonging to each age group, gender, region, and (in eligible waves) by level of psychological distress.

### Ethics approval

Ethical approval for the STS was granted originally by the UCL Ethics Committee (ID 0498/001). Participants provide informed consent to take part in the study, and all methods are carried out in accordance with relevant regulations. The data are not collected by UCL and are anonymised when received by UCL.

### Role of the funding source

The funders played no role in the study design, data collection, data analysis, interpretation, or writing of the report.

## Results

Of the 173,248 participants, 33,989 (19.6%) were current smokers, 31,446 (18.2%) were former smokers, and 107,813 (62.2%) had never regularly smoked. Sample characteristics, overall and by smoking status, are presented in Supplementary Table S2.

### Time trends among all working-age adults

**Fig. 1:**
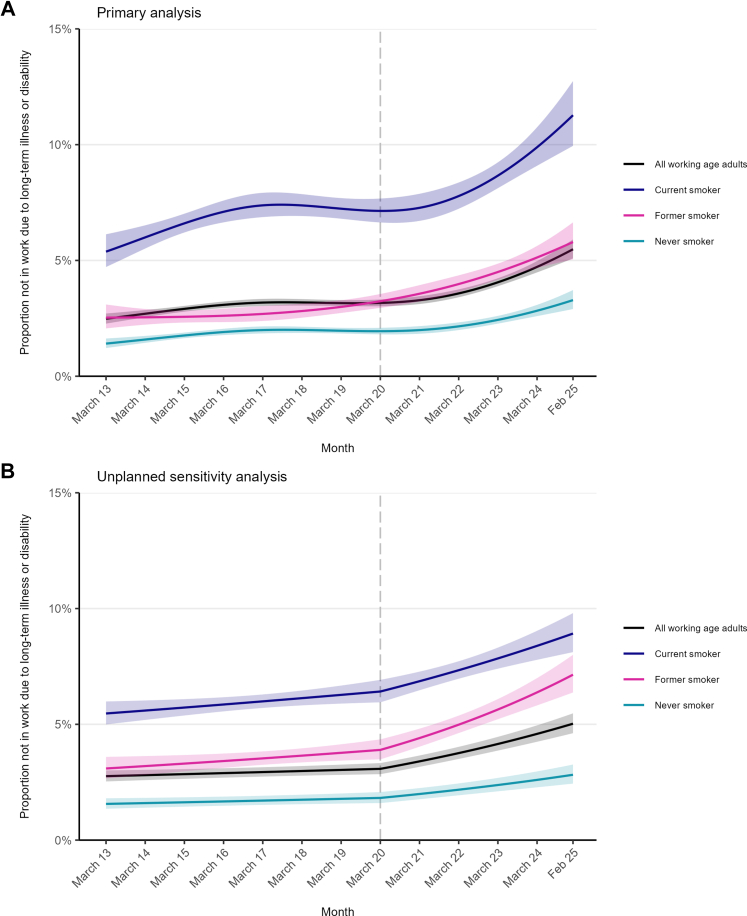
**Trends in health-related economic inactivity among working-age adults in England, overall and by smoking status, March 2013–February 2025.** Panel A shows results of the primary model, which modelled survey wave non-linearly using restricted cubic splines (see Supplementary Table S1 for model selection). Panel B shows the results of an unplanned sensitivity analysis, which used a segmented regression approach to model associations of the onset of the COVID-19 pandemic with a change in the trend of health-related economic inactivity. Lines represent modelled weighted prevalence by monthly survey wave, adjusted for age and gender (and, in Panel B, seasonality). Shaded bands represent 95% confidence intervals. The vertical dashed line indicates the timing of the onset of the COVID-19 pandemic in March 2020.

Across the study period, the prevalence of health-related economic inactivity in all working-age adults more than doubled, rising from 2.5% [2.3–2.7%] in March 2013 to 5.5% [5.1–5.9%] in February 2025 (PR = 2.21 [1.96–2.49]; Table 1). This increase was non-linear (Fig. 1A), with most of the growth occurring after the start of the pandemic (prevalence in March 2020: 3.2% [3.0–3.3%]; Supplementary Table S3).

**Table 1:** Modelled estimates of changes in the proportion of working-age adults in England not in work due to long-term illness or disability.

	% [95% CI] not in work due to long-term illness or disability^a^		Absolute percentage point change [95% CI]^b^	Relative change, prevalence ratio [95% CI]^c^
	March 2013	February 2025		
All working-age adults	2.5 [2.3–2.7]	5.5 [5.1–5.9]	3.0 [2.5–3.5]	2.21 [1.96–2.49]
By smoking status				
Never	1.4 [1.2–1.6]	3.3 [2.9–3.7]	1.9 [1.4–2.4]	2.33 [1.89–2.89]
Former	2.5 [2.1–3.1]	5.8 [5.0–6.6]	3.3 [2.3–4.2]	2.29 [1.78–2.98]
Current	5.4 [4.7–6.1]	11.3 [9.9–12.7]	5.9 [4.2–7.5]	2.10 [1.71–2.52]
By duration of abstinence, among former smokers^d^				
1 year	4.7 [3.4–6.5]	12.1 [9.6–15.1]	7.4 [4.8–10.8]	2.54 [1.85–3.82]
5 years	3.8 [3.0–4.7]	9.2 [8.0–10.7]	5.5 [4.2–7.1]	2.45 [2.00–3.16]
10 years	3.0 [2.3–3.8]	6.8 [5.7–8.0]	3.8 [2.7–5.0]	2.28 [1.82–3.03]
20 years	2.3 [1.7–3.1]	4.6 [3.8–5.7]	2.3 [1.4–3.4]	2.01 [1.53–2.85]

CI, confidence interval.

a Data are weighted estimates of prevalence in the first and last months in the study period, from logistic regression with survey month modelled non-linearly using restricted cubic splines (Supplementary Table S1 for model selection), adjusted for age and gender. Modelled estimates in each year are provided in Supplementary Table S3.

b Absolute percentage point change calculated as prevalence in February 2025 minus prevalence in March 2013 with 95% CIs calculated using bootstrapping (1000 replications).

c Prevalence ratio calculated as prevalence in February 2025 divided by prevalence in March 2013 with 95% CIs calculated using bootstrapping (1000 replications).

d Modelled estimates are shown for selected durations of abstinence to illustrate differences. Note that the model used to derive these estimates included data from former smokers with any duration of abstinence, not only those abstinent for 1, 5, 10, or 20 years.

The unplanned segmented regression analysis was consistent with this rise being associated with the onset of the pandemic (Fig. 1B, Supplementary Table S4). Before March 2020, the prevalence of health-related economic inactivity increased by 1.6% per year. This trend increased sharply after the pandemic started, with prevalence rising by 10.5% per year since. Note these percentages represent the yearly relative rather than absolute percentage point increase.

### Time trends by smoking status

At all timepoints, the prevalence of health-related economic inactivity was highest among current smokers and lowest among never smokers (Fig. 1). While relative increases in prevalence over time were broadly similar across smoking statuses, absolute increases were largest among current smokers (+5.9 percentage points [ppts]) compared with former (+3.3 ppts) and never smokers (+1.9 ppts; Table 1). By February 2025, 11.3% of working-age current smokers were not in work due to long-term illness or disability—nearly double the rate among former smokers (5.8%) and more than three times that of never smokers (3.3%).

When extrapolated to the national population, these figures suggest that approximately 700,000 current smokers were economically inactive due to ill health in early 2025 (34.9 million working-age adults x 17.8% current smoking prevalence x 11.3%), up from around 390,000 in 2013 (33.1 million x 21.8% × 5.4%). This is despite the overall decline in national smoking prevalence. The equivalent numbers for all working-age adults were 1.9 million in early 2025 (34.9 million x 5.5%), up from 830,000 in 2013 (33.1 million x 2.5%).

Exploratory analyses stratified by age group showed the prevalence of health-related economic inactivity was higher, and absolute increases over time were greater, with increasing age (Supplementary Fig. S2). For example, prevalence increased from 4.7% in March 2013 to 11.0% in February 2025 (+6.3 ppts) among those aged 55–64 compared with 0.7%–1.8% (+1.1 ppts) among those aged 18–24 (Supplementary Table S5). In addition, differences by smoking status were much more pronounced in older age groups (Supplementary Fig. S2, Supplementary Table S5).

### Time trends by duration of abstinence among former smokers

**Fig. 2:**
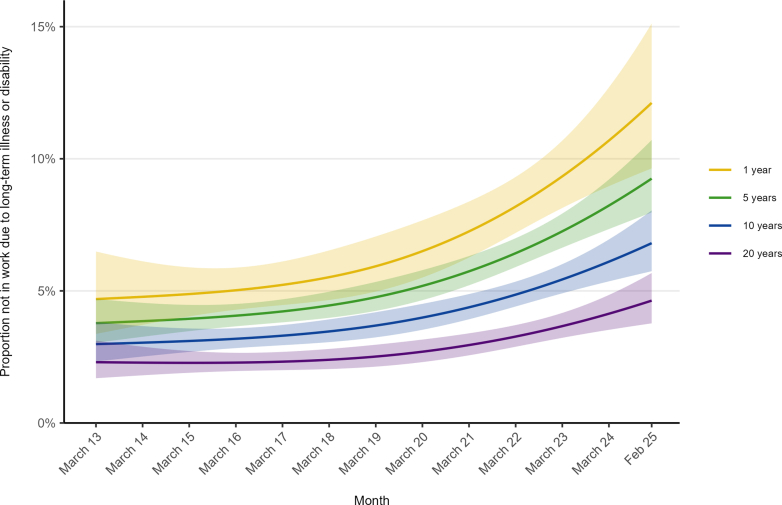
**Trends in health-related economic inactivity among working-age former smokers in England by duration of abstinence, March 2013–February 2025.** Lines represent modelled weighted prevalence by monthly survey wave and duration of abstinence, both modelled non-linearly using restricted cubic splines (see Supplementary Table S1 for model selection), adjusted for age and gender. Shaded bands represent 95% confidence intervals. Modelled estimates are shown for selected durations of abstinence to illustrate differences. Note that the model used to derive these estimates included data from former smokers with any duration of abstinence, not only those abstinent for 1, 5, 10, or 20 years.

Among former smokers, the prevalence of health-related economic inactivity was consistently higher among those with shorter durations of abstinence (Fig. 2). Absolute increases over time were largest among more recent quitters—for example, +7.4 ppts among those who quit one year ago compared with +2.3 ppts among those who quit 20 years ago—though relative increases were similar across durations of abstinence (Table 1).

### Sociodemographic profile of those not in work due to long-term illness or disability

In 2022–2025, current smokers who were not in work due to long-term illness or disability were on average 45.5 years old; one year younger than those who had never regularly smoked and three years younger than former smokers (Table 2). This pattern reflects broader age differences by smoking status (Supplementary Table S2). Notably, a quarter (25.2%) of current smokers not in work due to long-term illness or disability were aged under 35. More than half of those not in work due to long-term illness or disability were women, including 56.2% of current smokers (Table 2), consistent with overall gender distributions by smoking status (Supplementary Table S2). The proportion who described their gender in another way (i.e., not as a man or woman) was lowest among current smokers. There were no notable differences in the regional distribution of those not in work due to long-term illness or disability by smoking status (Table 2).

**Table 2:** Sociodemographic and psychological profile of working-age adults not in work due to long-term illness or disability in 2022–2025, overall and by smoking status.

	Not in work due to long-term illness or disability, % [95% CI]^a^			
	All working-age adults	Never smokers	Former smokers	Current smokers
Unweighted *N*	2254	809	672	773
Age (years)				
Mean (SD)	46.8 (12.2)	46.5 (12.3)	48.5 (12.1)	45.5 (12.0)
18–24	5.3 [4.2–6.3]	5.8 [3.9–7.6]	4.6 [2.8–6.3]	5.3 [3.6–7.0]
25–34	16.3 [14.6–18.0]	15.9 [13.0–18.7]	12.7 [9.8–15.6]	19.9 [16.9–23.0]
35–44	18.5 [16.8–20.3]	19.3 [16.3–22.3]	17.5 [14.3–20.7]	18.6 [15.6–21.6]
45–54	26.6 [24.7–28.6]	26.4 [23.0–29.7]	25.5 [22.0–29.0]	27.9 [24.5–31.3]
55–64	33.3 [31.2–35.3]	32.7 [29.3–36.1]	39.7 [35.8–43.6]	28.3 [25.0–31.5]
Gender				
Man	39.5 [37.3–41.7]	38.8 [35.1–42.4]	36.7 [32.8–40.6]	42.7 [38.9–46.4]
Woman	58.2 [56.0–60.4]	57.9 [54.2–61.6]	60.8 [56.9–64.8]	56.2 [52.4–59.9]
In another way	2.3 [1.7–2.9]	3.3 [2.1–4.5]	2.5 [1.3–3.6]	1.2 [0.5–1.9]
Region				
North East	5.8 [4.8–6.9]	5.6 [3.7–7.5]	6.2 [4.2–8.1]	5.8 [4.1–7.5]
North West	14.7 [13.1–16.4]	14.7 [11.9–17.5]	14.6 [11.8–17.5]	14.9 [12.2–17.6]
Yorkshire and the Humber	11.5 [10.1–12.9]	11.1 [8.7–13.4]	13.7 [10.9–16.4]	10.0 [7.8–12.2]
East Midlands	9.9 [8.6–11.2]	10.4 [8.1–12.7]	9.6 [7.3–12.0]	9.5 [7.3–11.8]
West Midlands	11.0 [9.6–12.4]	13.1 [10.6–15.6]	8.0 [5.8–10.1]	11.4 [9.0–13.8]
East of England	11.1 [9.7–12.5]	11.1 [8.9–13.3]	12.0 [9.3–14.7]	10.4 [8.1–12.7]
London	11.4 [10.0–12.7]	11.7 [9.4–14.0]	9.7 [7.4–12.0]	12.5 [10.1–14.9]
South East	14.1 [12.5–15.7]	12.0 [9.6–14.4]	17.4 [14.2–20.7]	13.3 [10.8–15.9]
South West	10.5 [9.2–11.8]	10.2 [8.0–12.4]	8.8 [6.5–11.1]	12.2 [9.8–14.7]
Past-month psychological distress^b^				
None/low	23.8 [21.3–26.4]	29.3 [24.7–33.9]	21.8 [17.2–26.4]	19.7 [15.7–23.7]
Moderate	35.9 [33.0–38.8]	36.9 [32.0–41.9]	38.3 [32.8–43.8]	32.8 [28.0–37.6]
Severe	40.2 [37.3–43.2]	33.7 [28.9–38.5]	39.9 [34.4–45.4]	47.5 [42.4–52.5]

a Data are presented as weighted column percentages with 95% confidence intervals, unless otherwise specified.

b Data on psychological distress were only collected in selected waves (January 2022–June 2023, January–March 2024, and January–February 2025); data shown are valid percentages based on participants not in work due to long-term illness or disability surveyed in these waves (all working-age adults *n* = 1212; never smokers *n* = 446; former smokers *n* = 349; current smokers *n* = 417).

Among those not in work due to long-term illness or disability surveyed in waves that assessed past-month psychological distress, the proportion reporting severe distress was higher among current smokers (47.5%) than former (39.9%) or never smokers (33.7%; Table 2). Overall, the proportion who reported severe distress was higher among those aged 18–34 (51.9% [45.1–58.8%]) compared with those aged ≥35 (36.9% [33.7–40.2%]).

## Discussion

This study highlights the extent to which smoking is associated with health-related economic inactivity. Consistent with official data from other national surveys conducted by the ONS,^3^, ^4^, ^5^ we observed a substantial increase in the proportion of working-age adults in England out of work due to long-term illness or disability, with rates more than doubling between 2013 and 2025—particularly since the start of the COVID-19 pandemic. However, our estimate of the number of working-age adults this represents (1.9 million, as of February 2025) was somewhat lower than the ONS data suggest (2.7 million).^1^ The prevalence of health-related economic inactivity was consistently highest—and absolute increases were largest—among current smokers, which meant the absolute disparity between current smokers and former and never smokers widened over time. By February 2025, one in nine working-age adults in England who smoked was not in work due to long-term illness or disability—approximately 700,000 people—of whom around a quarter were under the age of 35. This figure was up from approximately 390,000 in 2013, despite a decline in smoking prevalence across the period, due to increases in both the total population size and the proportion not in work due to long-term illness or disability.

While these findings highlight a strong association between smoking and health-related economic inactivity, they must be understood within the broader context of systemic inequality. Economic inactivity is shaped by a broad range of factors including poverty, education, geography, and access to healthcare and employment.^2^^,^^9^ Smoking prevalence is disproportionately concentrated in socioeconomically disadvantaged groups,^25^^,^^26^ among whom the burden of poor health and labour market exclusion is also greatest.^27^ In addition, selection bias within the group of current smokers may further strengthen the observed associations. In a context where smoking is increasingly discouraged, those who continue to smoke are more likely to be individuals facing multiple disadvantages, including greater addiction severity, unsuccessful quit attempts, and existing chronic health conditions. This results in a concentration of poorer health outcomes within the smoking population, potentially contributing to stronger associations with health-related economic inactivity. As such, the observed association between smoking and economic inactivity may, at least in part, reflect underlying social and structural disadvantage that simultaneously drives both health behaviours and health outcomes.

Differences by smoking status were more pronounced in older age groups, consistent with evidence that the health impacts of smoking typically become more evident in mid- and later life.^38^^,^^39^ In addition, in line with evidence showing benefits of smoking cessation for health and wellbeing,^38^^,^^39^ former smokers were less likely than current smokers to be economically inactive due to ill health. We observed a clear dose-response relationship among former smokers, with health-related economic inactivity most prevalent among recent quitters and decreasing with longer durations of abstinence. The higher prevalence of inactivity among recent quitters likely reflects, at least in part, the tendency for people to stop smoking following the onset or diagnosis of serious health conditions.^40^ While the dose-response pattern aligns with established health benefits of sustained cessation,^39^ it is important to recognise that it may also reflect underlying socioeconomic factors. Individuals who quit earlier are often more socioeconomically advantaged, and have better baseline health and greater access to cessation support^26^—factors that themselves contribute to lower risk of long-term economic inactivity. Consequently, duration of abstinence may serve as a proxy for broader socioeconomic position rather than a direct causal link between quitting smoking and re-entry into the labour market. This complexity underscores that while quitting smoking is beneficial, it does not necessarily translate into quick labour market reintegration.

The sociodemographic profile of people affected by health-related economic inactivity was broadly similar by smoking status. Those who currently smoked were slightly younger than former and never smokers, suggesting that smoking may be contributing to earlier disengagement from the workforce. Given the long-term nature of the health problems associated with smoking and socioeconomic disadvantage, this could leave people at risk of perpetual exclusion from economic opportunities. However, we note that across all smoking statuses, a substantial proportion of those not in work due to long-term illness or disability were aged under 35. Among this younger cohort, psychological distress may be an important factor. Previous studies have documented a sharp rise in distress among young adults in recent years,^10^ and in our analysis of survey waves that included data on past-month psychological distress, half of adults aged under 35 who were not in work due to long-term illness or disability reported experiencing severe distress (a higher rate than among those aged 35 or older). Levels of distress were also notably higher among current smokers than never smokers.^10^ These findings highlight the significant mental health burden among economically inactive young adults and people who smoke. The extent to which this a cause or consequence of economic inactivity is unclear and warrants further investigation. However, the relationship between psychological distress, smoking, and economic inactivity is likely bidirectional and rooted in structural disadvantage. Mental health problems may both contribute to and result from workforce exclusion and financial insecurity.^41^, ^42^, ^43^ Smoking, in turn, may be used as a coping mechanism in the context of chronic stress.^44^ These dynamics reinforce the need to understand economic inactivity not merely as an outcome of poor health behaviours, but as a social and public health issue shaped by broader inequality.

Our findings suggest that people transitioning away from smoking—particularly those managing chronic health conditions—may benefit from additional support to quit smoking and stay quit to improve their long-term employment prospects. More broadly, better support for people managing chronic health conditions while working—including workplace accommodations and occupational health interventions—may help reduce the risk of workforce dropout and improve long-term employment outcomes.^45^

These results also provide valuable evidence for economic modelling aimed at assessing the broader economic impact of smoking. Economic models that estimate the cost of smoking-related health issues—such as lost productivity, premature mortality, and healthcare expenditures—rely on high-quality, up-to-date data such as those provided in this study. By quantifying the extent of smoking-related economic inactivity, this study offers key inputs that can inform public health policies and tobacco control strategies. Specifically, the data underscore the need to strengthen tobacco control legislation and continue investing in population-level interventions and individual-level support for smoking cessation as part of broader efforts to systemically reduce health inequalities and long-term sickness-related unemployment and improve overall workforce participation.^46^

Strengths of this study include the large, representative sample and monthly time series, which support the generalisability of the findings to the adult population in England. A key limitation is that the cross-sectional design limits causal inference. While associations between smoking and inactivity are observed, the direction of causality (e.g., whether smoking leads to inactivity or inactivity prompts smoking) cannot be definitively established. There is a vast literature showing that smoking is causally linked to a range of serious diseases, causing long-term ill health and disability.^47^ In addition, both the dose-response association we observed between duration of abstinence and health-related economic inactivity among former smokers, and the fact we observed greater differences by smoking status among older age groups (who would have longer smoking histories), are consistent with a causal relationship. However, these patterns could also be explained by socioeconomic inequalities that shape both health behaviours and outcomes.

Data were not collected on the specific reason for health-related economic inactivity, so we were unable to explore the contribution of different smoking-associated diseases or mental health concerns to workforce dropout. Nor did we adjust for macroeconomic factors such as gross domestic product or labour market conditions, which may contribute to health-related inactivity. Future work could explore these influences in more detail. We used a hybrid sampling approach rather than random probability sampling, although comparisons with other sources suggest the survey recruits a nationally-representative sample and produces similar estimates of key smoking variables. Data were not consistently collected on ethnicity so we were unable to explore differences between ethnic groups. While our findings are most directly relevant to the UK context, similar trends may be observable in other high-income countries with comparable smoking prevalence, healthcare access, and labour market conditions, warranting further international comparison.

In conclusion, current smoking is strongly associated with health-related economic inactivity, and this disparity has widened over time in absolute terms. These associations are embedded in broader patterns of structural inequality that shape both health behaviours and labour market outcomes. Efforts to reduce smoking prevalence may contribute to tackling rising inactivity and improving labour market participation, but addressing this issue at scale will also require systemic action to improve the social, economic, and structural conditions that people who smoke live within that make both smoking and work participation harder to address.

## Contributors

SEJ, SC, and JB conceived and designed the study. SEJ analysed the data and wrote the first draft. All authors provided critical revisions. SEJ and JB accessed and verified the data. All authors had full access to the data and were responsible for the decision to submit the manuscript.

## Data sharing statement

The data used in these analyses are available on Open Science Framework (https://osf.io/nygzr/), with age provided in bands to preserve anonymity.

## Declaration of interests

SJ and SC receive salary support from Cancer Research UK. JB and SC are members of the Behavioural Research UK Leadership Hub which is supported by the Economic and Social Research Council. SJ is President and SC is Past President of the Society for Research on Nicotine and Tobacco—Europe. SJ has received payment from the English Department of Health and Social Care (via Freuds Communication Agency) to undertake research to support public health mass media campaigns. JB has received unrestricted research funding from Pfizer and J&J, who manufacture smoking cessation medications, and royalties from Wiley for Theory of Addiction (second edition). All authors declare no financial links with tobacco companies, e-cigarette manufacturers, or their representatives.
