# Supplementary material for: Trends in health-related economic inactivity by smoking status in England, 2013–2025: a population-based analysis
Source: Lancet Reg Health Eur. 2025 Sep 7;57:101419. doi: 10.1016/j.lanepe.2025.101419 (PMC12541643; doi:10.1016/j.lanepe.2025.101419)
Supplement: Supplementary Figs. S1 and S2 and Tables S1–S4 [file mmc1.pdf]

Table S1. Model selection: AIC values for models with 3, 4, and 5 knots

	AIC		
	**3 knots**	**4 knots**	**5 knots**
All working age adults	55115.78	55092.77	55093.90
By smoking status	53041.24	53020.42	53025.04
By duration of abstinence, among former smokers	10066.29	10067.57	10072.18

AIC, Akaike Information Criterion.
Shaded cells indicate the best fitting model (the model with the lowest AIC or the simplest model within 2 AIC units).

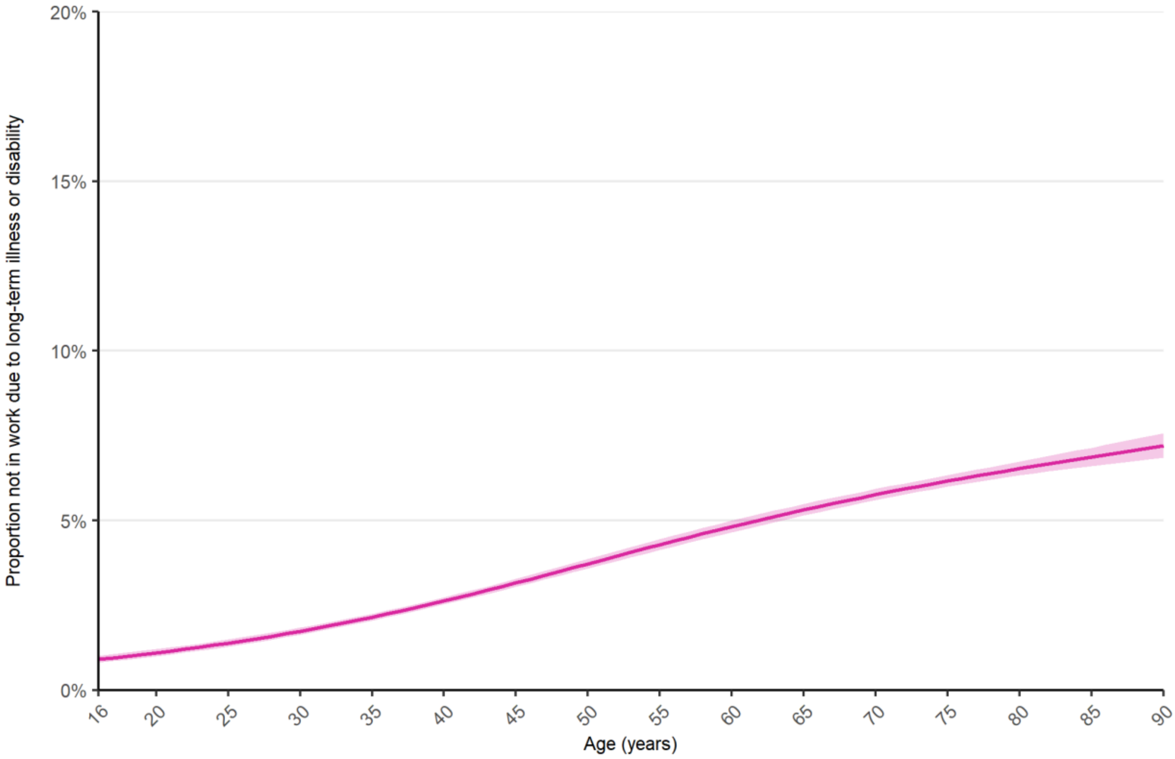

Figure S1. Association between age and health-related economic inactivity. The line represents modelled weighted prevalence of health-related economic inactivity by age, modelled non-linearly using restricted cubic splines (three knots). Shaded band represents the 95% confidence interval.

Table S2. Sample characteristics

	All working-age adults	Never smokers	Former smokers	Current smokers
Unweighted *N*	173,248	107,813	31,446	33,989
Age (years)				
Mean (SD)	40.5 (13.4)	39.9 (13.6)	44.9 (12.4)	38.3 (13.1)
18-24	15.9	17.8	6.6	18.6
25-34	22.1	21.8	17.7	26.9
35-44	21.0	20.8	22.4	20.4
45-54	22.1	21.4	26.8	19.8
55-64	18.9	18.2	26.5	14.3
Gender				
Man	49.8	47.9	51.4	54.3
Woman	49.8	51.7	48.3	45.2
In another way	0.4	0.3	0.3	0.5
Region				
North East	5.0	4.6	5.7	5.5
North West	13.2	13.0	12.8	14.5
Yorkshire and the Humber	10.2	9.9	10.3	10.7
East Midlands	8.7	8.6	8.5	9.0
West Midlands	10.3	10.6	9.7	9.8
East of England	10.9	11.0	11.0	10.7
London	16.9	18.6	13.1	15.3
South East	15.4	15.0	17.7	14.7
South West	9.4	8.7	11.2	9.9

Data are presented as weighted column percentages, unless otherwise specified.

Table S3. Modelled estimates of changes in the proportion of working-age adults in England not in work due to long-term illness or disability

	% [95%CI] not in work due to long-term illness or disability[1]												
	**March 2013**	**March 2014**	**March 2015**	**March 2016**	**March 2017**	**March 2018**	**March 2019**	**March 2020**	**March 2021**	**March 2022**	**March 2023**	**March 2024**	**February 2025**
All working-age adults	2.5 [2.3–2.7]	2.7 [2.5–2.9]	2.9 [2.8–3.0]	3.1 [2.9–3.2]	3.2 [3.0–3.3]	3.2 [3.0–3.3]	3.2 [3.0–3.3]	3.2 [3.0–3.3]	3.3 [3.1–3.5]	3.6 [3.4–3.8]	4.1 [3.9–4.2]	4.7 [4.5–5.0]	5.5 [5.1–5.9]
By smoking status													
Never	1.4 [1.2–1.6]	1.6 [1.4–1.7]	1.8 [1.6–1.9]	1.9 [1.8–2.0]	2.0 [1.8–2.2]	2.0 [1.9–2.1]	2.0 [1.8–2.1]	1.9 [1.8–2.1]	2.0 [1.8–2.2]	2.2 [2.0–2.3]	2.4 [2.3–2.6]	2.8 [2.6–3.1]	3.3 [2.9–3.7]
Former	2.5 [2.1–3.1]	2.5 [2.2–2.9]	2.6 [2.3–2.8]	2.6 [2.3–2.9]	2.7 [2.4–3.0]	2.8 [2.5–3.1]	3.0 [2.7–3.3]	3.2 [3.0–3.6]	3.6 [3.2–3.9]	4.0 [3.6–4.4]	4.5 [4.2–4.9]	5.1 [4.7–5.6]	5.8 [5.0–6.6]
Current	5.4 [4.7–6.1]	6.0 [5.5–6.5]	6.6 [6.2–7.0]	7.1 [6.6–7.6]	7.4 [6.9–7.9]	7.4 [6.9–7.9]	7.2 [6.8–7.7]	7.1 [6.6–7.7]	7.3 [6.7–7.9]	7.8 [7.2–8.4]	8.7 [8.1–9.2]	9.9 [9.1–10.8]	11.3 [9.9–12.7]
By duration of abstinence, among former smokers[2]													
1 year	4.7 [3.4–6.5]	4.8 [3.7–6.1]	4.9 [4.0–5.9]	5.0 [4.3–5.9]	5.2 [4.5–6.1]	5.5 [4.7–6.5]	5.9 [5.0–7.1]	6.5 [5.5–7.7]	7.3 [6.3–8.4]	8.2 [7.2–9.3]	9.3 [8.1–10.7]	10.7 [9.0–12.7]	12.1 [9.6–15.1]
5 years	3.8 [3.0–4.7]	3.9 [3.3–4.5]	3.9 [3.5–4.5]	4.1 [3.7–4.5]	4.2 [3.8–4.7]	4.4 [4.0–5.0]	4.8 [4.2–5.3]	5.2 [4.6–5.8]	5.7 [5.2–6.3]	6.4 [5.9–7.0]	7.3 [6.6–7.9]	8.2 [7.3–9.2]	9.2 [8.0–10.7]
10 years	3.0 [2.3–3.8]	3.0 [2.5–3.7]	3.1 [2.7–3.6]	3.2 [2.8–3.6]	3.3 [2.9–3.7]	3.5 [3.1–3.9]	3.7 [3.2–4.2]	4.0 [3.5–4.5]	4.4 [3.9–4.9]	4.9 [4.4–5.3]	5.4 [4.9–6.0]	6.1 [5.4–6.9]	6.8 [5.7–8.0]
20 years	2.3 [1.7–3.1]	2.3 [1.8–2.9]	2.3 [1.9–2.7]	2.3 [2.0–2.7]	2.3 [2.0–2.7]	2.4 [2.0–2.8]	2.5 [2.1–3.0]	2.7 [2.3–3.2]	2.9 [2.6–3.4]	3.3 [2.9–3.7]	3.7 [3.2–4.2]	4.1 [3.5–4.8]	4.6 [3.8–5.7]

[1] Data are weighted estimates of prevalence from logistic regression with survey month modelled non-linearly using restricted cubic splines (**Table S1** for model selection), adjusted for age and gender.

[2] Modelled estimates are shown for selected durations of abstinence to illustrate differences. Note that the model used to derive these estimates included data from former smokers with any duration of abstinence, not only those abstinent for 1, 5, 10, or 20 years.

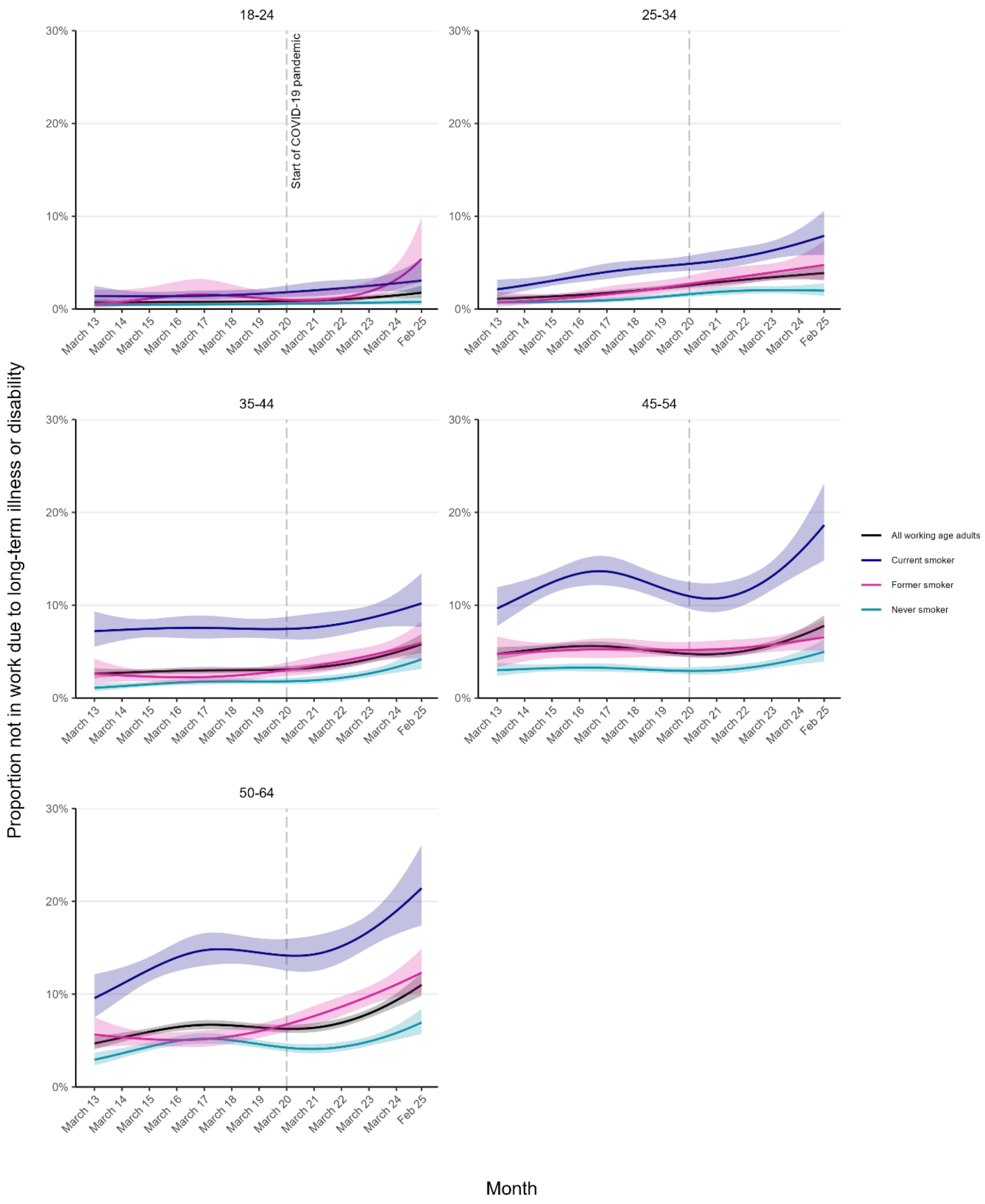

Figure S2. Age-stratified trends in health-related economic inactivity among working-age adults in England, overall and by smoking status, March 2013 to February 2025. Lines represent modelled weighted prevalence within each age group by monthly survey wave, modelled non-linearly using restricted cubic splines (four knots), adjusted for age and gender. Shaded bands represent 95% confidence intervals.

Table S4. Segmented regression results: changes in annual trends in the proportion of working-age adults in England not in work due to long-term illness or disability since the onset of the COVID-19 pandemic in March 2020

	**All working-age adults**	**Never smokers**	**Former smokers**	**Current smokers**
**Annual trends, RR [95% CI]**				
Pre-announcement trend (Mar 13 – Mar 20)	1.016 [1.003-1.029]	1.022 [1.001-1.044]	1.033 [1.004-1.064]	1.023 [1.004-1.043]
Change in annual trend (Mar 20)	1.088 [1.059-1.118]	1.069 [1.023-1.118]	1.095 [1.034-1.158]	1.045 [1.002-1.090]
Post-announcement trend (Mar 20 – Feb 25)	1.105 [1.073-1.138]	1.093 [1.041-1.148]	1.131 [1.062-1.206]	1.069 [1.021-1.120]
**Predicted prevalence estimates, % [95% CI]**				
March 2013	2.8 [2.5-3.0]	1.6 [1.4-1.8]	3.1 [2.7-3.6]	5.5 [5.0-6.0]
March 2020	3.1 [2.8-3.3]	1.8 [1.6-2.1]	3.9 [3.5-4.4]	6.4 [6.0-6.9]
February 2025	5.0 [4.6-5.5]	2.8 [2.4-3.3]	7.1 [6.4-8.0]	8.9 [8.1-9.8]

RR, risk ratio; CI, confidence interval.

Results shown are derived from segmented regression analyses (using generalised additive models) of data collected from March 2013 to February 2025. Models are adjusted for age, gender, and seasonality.

Table S5. Age-specific modelled estimates of changes in the proportion of working-age adults in England not in work due to long-term illness or disability

	% [95%CI] not in work due to long-term illness or disability[1]		Absolute percentage point change [95% CI][2]	Relative change, prevalence ratio [95% CI][3]
	**March 2013**	**February 2025**		
All working-age adults				
18-24	0.7 [0.5–1.0]	1.8 [1.2–2.5]	1.1 [0.4-1.9]	2.54 [1.41-4.56]
25-34	1.1 [0.8–1.5]	3.9 [3.2–4.7]	2.8 [1.9-3.7]	3.56 [2.47-5.13]
35-44	2.6 [2.1–3.2]	5.8 [4.9–6.9]	3.2 [2.0-4.4]	2.22 [1.68-2.92]
45-54	4.7 [4.1–5.5]	7.8 [6.8–8.9]	3.0 [1.7-4.4]	1.64 [1.31-2.05]
55-64	4.7 [4.1–5.4]	11.0 [9.8–12.3]	6.3 [4.9-7.9]	2.34 [1.92-2.85]
Never smokers				
18-24	0.5 [0.2–0.9]	0.8 [0.4–1.4]	0.3 [0.0-0.9]	1.70 [0.61-4.34]
25-34	0.7 [0.4–1.1]	2.0 [1.4–2.8]	1.3 [0.5-2.1]	2.78 [1.56-5.50]
35-44	1.1 [0.7–1.6]	4.2 [3.1–5.6]	3.1 [1.7-4.4]	3.82 [2.16-6.55]
45-54	3.0 [2.4–3.8]	5.0 [3.9–6.3]	2.0 [0.5-3.6]	1.67 [1.15-2.38]
55-64	2.9 [2.3–3.7]	6.9 [5.7–8.4]	4.0 [2.5-5.6]	2.36 [1.74-3.22]
Former smokers				
18-24	0.5 [0.1–2.1]	5.4 [2.9–9.9]	4.9 [1.8-8.9]	10.4 [2.74-134]
25-34	0.7 [0.3–1.9]	4.7 [3.0–7.3]	4.1 [1.8-6.6]	6.88 [2.32-32.4]
35-44	2.6 [1.6–4.2]	5.9 [4.3–8.2]	3.3 [0.8-5.7]	2.27 [1.23-4.24]
45-54	4.7 [3.4–6.6]	6.6 [5.1–8.5]	1.8 [0.0-4.2]	1.39 [0.91-2.21]
55-64	5.6 [4.2–7.5]	12.3 [10.1–14.9]	6.7 [3.7-9.7]	2.18 [1.53-3.13]
Current smokers				
18-24	1.4 [0.8–2.5]	3.1 [1.7–5.4]	1.7 [0.0-3.9]	2.18 [0.91-5.14]
25-34	2.1 [1.4–3.2]	7.9 [5.8–10.6]	5.8 [3.3-8.7]	3.73 [2.15-6.19]
35-44	7.2 [5.5–9.3]	10.2 [7.6–13.5]	3.0 [0.0-6.9]	1.42 [0.94-2.18]
45-54	9.6 [7.8–11.9]	18.6 [14.8–23.2]	9.0 [4.0-13.4]	1.93 [1.35-2.63]
55-64	9.6 [7.5–12.1]	21.4 [17.4–26.1]	11.9 [7.0-17.0]	2.24 [1.62-3.14]

CI, confidence interval.

[1] Data are weighted estimates of prevalence in the first and last months in the study period, from logistic regression with survey month modelled non-linearly using restricted cubic splines (four knots), adjusted for age and gender.

[2] Absolute percentage point change calculated as prevalence in February 2025 minus prevalence in March 2013 with 95% CIs calculated using bootstrapping (1,000 replications).

[3] Prevalence ratio calculated as prevalence in February 2025 divided by prevalence in March 2013 with 95% CIs calculated using bootstrapping (1,000 replications).
